# Nanowarming of vitrified pancreatic islets as a cryopreservation technology for transplantation

**DOI:** 10.1002/btm2.10416

**Published:** 2022-09-27

**Authors:** Taisei Wakabayashi, Masahiro Kaneko, Tomoki Nakai, Masanobu Horie, Hiroyuki Fujimoto, Masazumi Takahashi, Shota Tanoue, Akira Ito

**Affiliations:** ^1^ Department of Chemical Systems Engineering, School of Engineering Nagoya University Nagoya Japan; ^2^ Radioisotope Research Center, Agency of Health, Safety and Environment Kyoto University Kyoto Japan; ^3^ Technical Department Dai‐Ichi High Frequency Co., Ltd Kawasaki Japan

**Keywords:** cell transplantation, cryopreservation, diabetes, inductive heating, islet, magnetic nanoparticle

## Abstract

Biobanking of pancreatic islets for transplantation could solve the shortage of donors, and cryopreservation of vitrified islets is a possible approach. However, a technological barrier is rewarming of large volumes both uniformly and rapidly to prevent ice formation due to devitrification. Here, we describe successful recovery of islets from the vitrified state using a volumetric rewarming technology called “nanowarming,” which is inductive heating of magnetic nanoparticles under an alternating magnetic field. Convective warming using a 37°C water bath as the gold standard for rewarming of vitrified samples resulted in a decrease in the viability of mouse islets in large volumes (>1 ml) owing to devitrification caused by slow warming. Nanowarming showed uniform and rapid rewarming of vitrified islets in large volumes. The viability of nanowarmed islets was significantly improved and islets transplanted into streptozotocin‐induced diabetic mice successfully lowered serum glucose. The results suggest that nanowarming will lead to a breakthrough in biobanking of islets for transplantation.

## 1 INTRODUCTION

Transplantation of organs and tissues saves millions of lives and improves the quality of life each year globally. However, the global shortage of organs and tissues for transplantation has been recognized as one of the greatest crises of biomedicine.^1^ A major limitation of transplantation is the ischemic injury in organs and tissues during preservation. The timeframe in which organs and tissues remain viable and functional under cold storage (generally on ice) is considerably short.^2^ Pancreases are used for transplants up to only 8–12 h,^3^ which limits transport of pancreases from donors to potential recipients at geographically remote locations.

A promising approach to biobanking of organs and tissues is ice‐free cryopreservation by vitrification using rapid cooling that exceeds the critical cooling rate (CCR; the rate necessary to avoid ice formation during cooling) to an extremely low temperature (≤130°C) below the glass transition.^4^ Cryopreservation by vitrification was originally developed to preserve cells and small/thin tissues such as embryos and ovaries in small volumes (generally hundreds of microliters) of cryoprotective agent (CPA).^5^, ^6^ While the CCR is relatively easy to achieve by direct immersion in liquid nitrogen (−196°C), the main barrier of cryopreservation is to achieve rapid and uniform rewarming that exceeds the critical warming rate (CWR; the rate necessary to avoid ice formation due to devitrification during rewarming), which is an order of magnitude higher than the CCR.^4^ The conventional method of rewarming is convective warming by immersing vitrified CPA in a water bath at 37°C until temperature at the center of CPA reaches 0°C, to avoid cellular or tissue damage caused by overheating and CPA toxicity, which leads to a low warming rate at the center of a large volume of CPA with large diameters. Additionally, high‐temperature gradients between the center and edge of the large volume of CPA cause thermal stress strain that leads to cracking during rewarming.^7^

Nanowarming is a cryopreservation technology for volumetric warming of vitrified CPAs.^8^, ^9^, ^10^, ^11^ Superparamagnetic nanoparticles generate heat under an alternating magnetic field by Brownian and Néelian relaxation.^12^, ^13^ In general, magnetite (Fe_3_O_4_) with a size of around 10 nm and magnetic field applicators that generate an alternating magnetic field with an order of tens of kA m^−1^ at several 100 kHz have been used.^8^, ^10^, ^14^ Magnetite nanoparticles are loaded in CPA (magnetite‐loaded CPA [mCPA]) and inductive heating of well‐dispersed magnetite nanoparticles enables uniform and rapid rewarming of vitrified CPA independently of the sample volume in theory. In 2016, Wang et al. first proposed nanowarming to vitrified human stem cells with high viability, intact stemness, and multilineage potential of differentiation.^15^ In 2017, the second applied nanowarming to vitrified fibroblasts, vessel segments and heart valve tissues.^8^ Our group reported that vitrified human‐induced pluripotent stem cells were successfully rewarmed by nanowarming.^9^ In these cases, cells and tissues were vitrified by immersion in an mCPA. More recently, a silica‐coated magnetic nanoparticle was reported to have sufficient dispersion stability for perfusion of rat kidneys.^11^ Chiu‐Lam et al. developed magnetic nanoparticles coated with a dense, covalently grafted brush of polyethylene glycol (PEG) for perfusion of rat hearts, and reported whole‐heart cryopreservation and nanowarming,^10^ which demonstrated the proof‐of‐concept of nanowarming for biobanking and transplantation. Although these reports showed recovery of cell viability and tissue histology, the functionality of the nanowarmed tissues and organs, which is the most important factor for transplants, remains to be demonstrated.

Islet transplantation has been an established therapy as a potential curative treatment for patients with insulin‐dependent diabetes and severe hypoglycemia.^16^ In addition to the shortage of donors, a major problem in the clinical transplantation of islets is the low yield of islets that can be obtained from one donor pancreas. Islets must be collected from different donors and preserved until sufficient islets are obtained. To overcome these issues, cryopreservation of islets will offer the potential to biobank islets for transplantation on demand.^17^, ^18^ Jutte et al. first reported that vitrified mouse^19^ and human^20^ islets in 1 ml CPAs were successfully rewarmed by convective warming. The mouse islets showed insulin secretion and the capability to lower blood glucose to normal levels after transplantation into streptozotocin (STZ)‐induced diabetic mice.^19^ In the current study, we apply nanowarming as a volumetric rewarming technology to islets cryopreservation for biobanking and clinical transplantation. Specifically, cell viability, glucose‐stimulated insulin release, and the capability to lower blood glucose levels after transplantation were investigated to demonstrate the feasibility of nanowarming for clinical islet transplantation.

## 2 RESULTS

### 2.1 Volume limitation of convective warming in rewarming of vitrified islets

**FIGURE 1:**
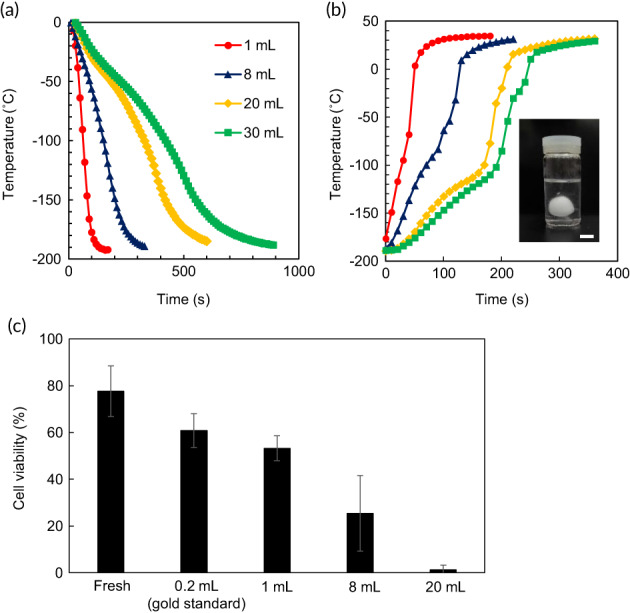
Convective warming of large volumes of VS55. Cooling for vitrification (a) and convective warming for rewarming (b) were carried out by immersing glass vials in liquid nitrogen and a 37°C water bath, respectively. Average data of three independent experiments of temperatures at the center of vials are shown. Red circles, 1 ml; blue triangles, 8 ml; yellow diamonds, 20 ml; green squares, 30 ml. The inset photograph in (b) shows an ice ball in 20 ml VS55 induced by devitrification during convective warming. Scale bar, 10 mm. (c) Effects of convective warming for rewarming on the viability of vitrified mouse islets. After vitrifying and convective rewarming, cell viability was assayed using a cell viability imaging kit based on Hoechst 33342 for live cells and SYTOX green nucleic acid stain for dead cells. Data are expressed as the mean ± SD of three independent experiments.

The commonly used CPA solution VS55, which has a CCR of 2.5°C min^−1^, a CWR of 50°C min^−1^, and a glass transition temperature of −123°C,^21^ was used in this study. Variously sized glass vials (Table S1) that contained VS55 (1–30 ml) were submersed in liquid nitrogen and the temperature at the center of the vials was monitored over time (Figure 1a). Temperature–time plots showed smooth curved lines during cooling for 1 and 8 ml, whereas inflection points (e.g., at around −50°C), which might suggest ice formation, were observed for 20 and 30 ml. However, all samples had a transparent glassy appearance at around −196°C, which indicated that VS55 was successfully vitrified at volumes smaller than 30 ml (16 mm radius). Table 1 shows the cooling rates and all samples (1, 8, 20, and 30 ml) achieved a CCR of 2.5°C min^−1^ by cooling in liquid nitrogen.

**TABLE 1:** Convective cooling and warming rates

Volume (radius)	Cooling rate (°C min^−1^)^a^	Warming rate (°C min^−1^)^a^
1 ml (r = 5 mm)	166.0 ± 11.9	163.2 ± 5.7
8 ml (r = 10 mm)	60.8 ± 5.3	58.8 ± 17.5
20 ml (r = 14 mm)	38.1 ± 5.5	26.5 ± 2.7
30 ml (r = 16 mm)	25.5 ± 4.3	23.0 ± 6.0

^a^ The cooling and warming rates at the center of vials were calculated by the slopes between −38°C (the melting temperature of VS55) and −123°C (the glass transition temperature of VS55). Data are the mean and SD of three independent experiments.

During the rewarming process (Figure 1b), convective warming resulted in devitrification of VS55, which was evidenced by ice formation in the sample larger than 20 ml (14 mm radius) by direct visual inspection (inset photograph of Figure 1b). During rewarming of 30‐ml samples, two typical changes of temperature in the time course were observed around −100 to −80°C and −50 to −30°C, which indicated the recrystallization temperature (−82°C) and the melting temperature (−38°C) of VS55.^21^ Warming rates of samples with 1‐, 8‐, 20‐, and 30‐ml volumes are shown in Table 1. Only the warming rate in 1 ml (5 mm radius) well exceeded the CWR, which indicated that convective warming using a water bath at 37°C was too slow to achieve the CWR in large‐volume CPAs (≥several milliliters).

Next, we measured the cell viability of vitrified islets after convective warming (Figure 1c) using the Sytox green/Hoechst 33342 assay.^9^ The cell viability of fresh islets isolated from mice by the collagenase digestion method was 77.7% ± 10.8%. For islet cryopreservation, 0.2 ml CPA in a 2‐ml polypropylene cryovial was used as a control (gold standard). The cell viability after rewarming by convective warming was 60.9% ± 7.2%, which was approximately 80% of the fresh islets. In the case of a small volume with 1 ml (warming rate, 163.2 ± 5.7°C min^−1^ [Table 1] >CWR), the cell viability (53.3% ± 5.3%) was comparable with the gold standard. For larger volumes, cell viability was decreased to 25.4% ± 16.1% for 8 ml (10 mm radius) and almost all cells had died in 20‐ml samples. These results indicate that a warming rate that exceeds the CWR is crucial for the viability of vitrified islets.

### 2.2 Characterization of warming rates by mCPA‐mediated nanowarming

**FIGURE 2:**
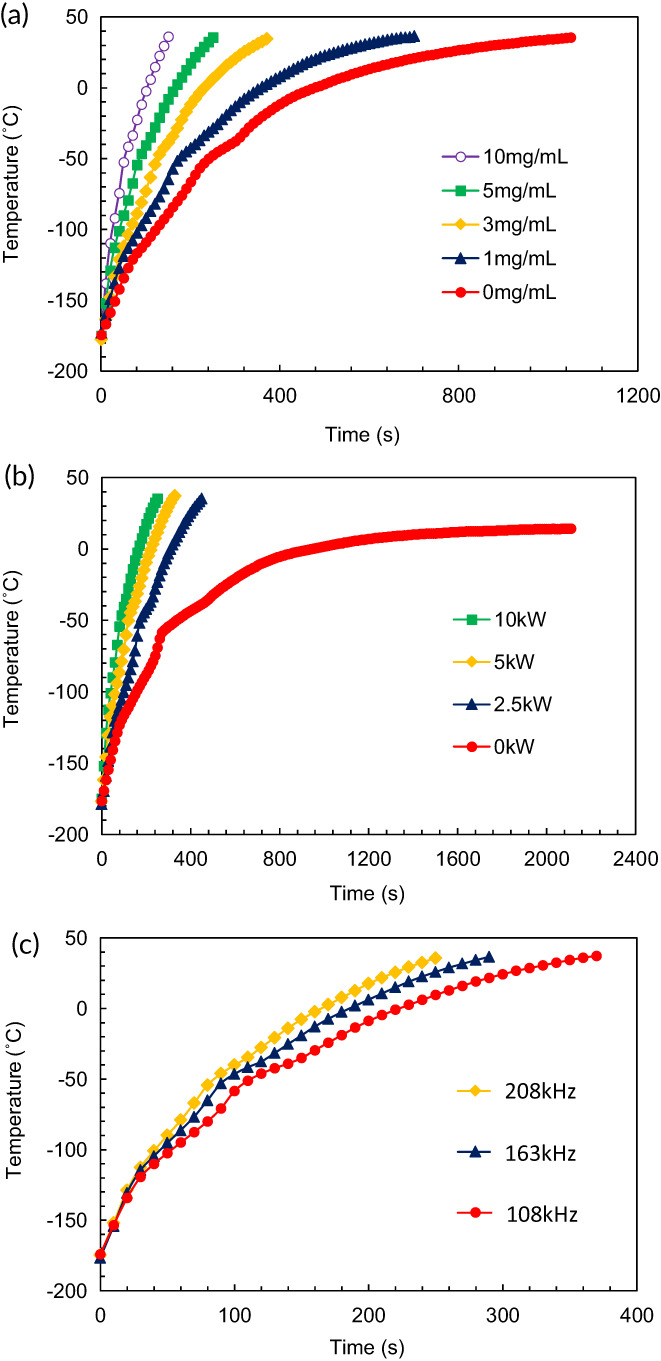
Temperature profiles of nanowarming. Nanowarming of vitrified VS55 was carried out by inductive heating of magnetite nanoparticles. Average data of three independent experiments of temperatures at the center of vials are shown. (a) Nanowarming power output, 10 kW; magnetite concentration, 0 mg ml^−1^ (red closed circles), 1 mg ml^−1^ (blue triangles), 3 mg ml^−1^ (yellow diamonds), 5 mg ml^−1^ (green squares), and 10 mg ml^−1^ (purple open circles); frequency, 208 kHz. (b) Nanowarming power output, 0 kW (red circles), 2.5 kW (blue triangles), 5 kW (yellow diamonds), and 10 kW (green squares); magnetite concentration, 5 mg ml^−1^; frequency, 208 kHz. (c) Nanowarming power output, 10 kW; magnetite concentration, 5 mg ml^−1^; frequency, 108 kHz (red circles), 163 kHz (blue triangles), and 208 kHz (yellow diamonds).

Using mCPA, nanowarming rates were evaluated in an alternating magnetic field (Figure 2). Magnetite nanoparticles of 10 nm in size, which were assumed to be superparamagnetic,^12^, ^13^ were well dispersed in VS55, and 1 ml vitrified mCPA (magnetite concentrations: 0, 1, 3, 5, and 10 mg ml^−1^) with liquid nitrogen was exposed to an alternating magnetic field (power outputs: 0, 2.5, 5, and 10 kW; frequencies: 108, 163, and 208 kHz). Temperature–time plots showed an inflection point at around −45°C during rewarming, especially under control conditions (magnetite concentrations: 0 mg ml^−1^ or power outputs: 0 kW), which may suggest ice formation. The warming rates increased with the magnetite concentration (Figure 2a), power output (Figure 2b), and frequency (Figure 2c), and the CWR was achieved at 5 mg ml^−1^ magnetite, 10 kW, and 208 kHz (warming rate, 72.0 ± 2.4°C min^−1^) (Table 2). In the following experiments, we performed nanowarming under these conditions.

**TABLE 2:** Characterization of nanowarming in 1 ml

Magnetite conc. (mg ml^−1^)	Power output (kW)	Frequency (kHz)	Warming rate (°C min^−1^)^a^
0	10	208	25.7 ± 1.0
1			33.5 ± 0.9
3			51.4 ± 4.7
5			72.0 ± 2.4
10			123.9 ± 3.0

Magnetite conc. (mg ml^−1^)	Power output (kW)	Frequency (kHz)	Warming rate (°C min^−1^)^a^
5	0	208	17.5 ± 0.7
	2.5		32.5 ± 1.4
	5		50.8 ± 1.2
	10		72.0 ± 2.4

Magnetite conc. (mg ml^−1^)	Power output (kW)	Frequency (kHz)	Warming rate (°C min^−1^)^a^
5	10	108	47.4 ± 1.4
		163	56.6 ± 2.2
		208	72.0 ± 2.4

^a^ The warming rates at the center of vials were calculated by the slopes between −38°C (the melting temperature of VS55) and − 123°C (the glass transition temperature of VS55). Data are the mean and SD of three independent experiments.

**FIGURE 3:**
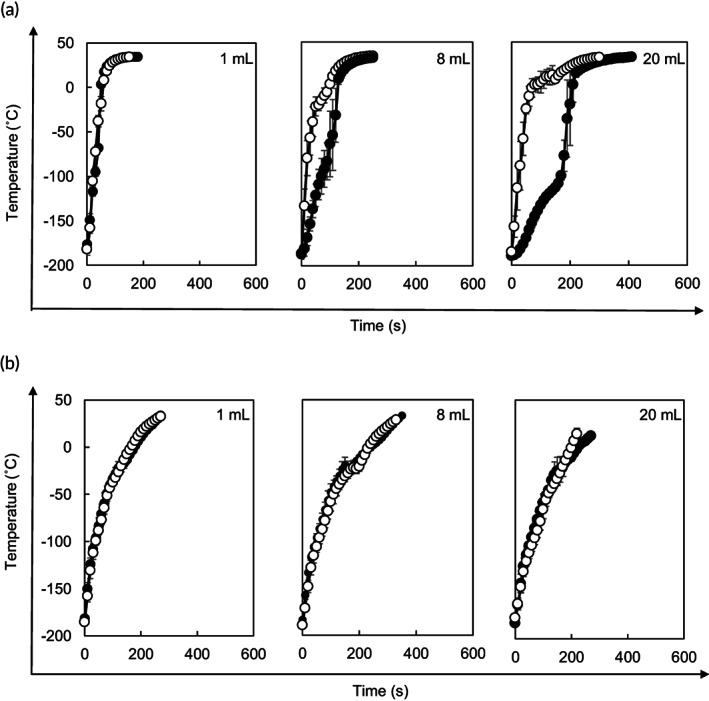
Scale‐up of convective warming and nanowarming. Convective warming (a) and nanowarming (5 mg magnetite ml^−1^ VS55, 10 kW and 208 kHz) (b) of vitrified VS55 in 1‐, 8‐, and 20‐ml volumes were carried out. Temperatures at the center (closed circles) and edge (open circles) of the vials were measured by optical fiber probes. Data are expressed as the mean ± SD of three independent experiments.

We next conducted scale‐up of the volume and monitored the time course of the temperature change at the center and edge of samples with 1‐, 8‐, and 20‐ml volumes during rewarming (Figure 3). In convective warming of CPA, the temperature rise at the center was delayed from the edge and gaps in temperature between the center and edge were evident in samples with 8‐ml (average maximal gradient [Δ*T*_max_], 99.5°C) and 20‐ml (Δ*T*_max_, 151.2°C) volumes (Figure 3a). During rewarming by convective warming, cracks were observed in 8 and 20 ml CPA (Figure S1) because their Δ*T*_max_ was well beyond the stress‐to‐fracture temperature limit of VS55 glass (38°C).^11^ However, nanowarming of mCPA showed no obvious temperature gradient between the center and edge of samples (Figure 3b) or obvious cracks in the samples. Importantly, the nanowarming rates were independent of the sample volume (Figure 3b). These results indicate that nanowarming is a scalable technique that enables uniform and rapid rewarming.

### 2.3 Nanowarming of pancreatic islets

**FIGURE 4:**
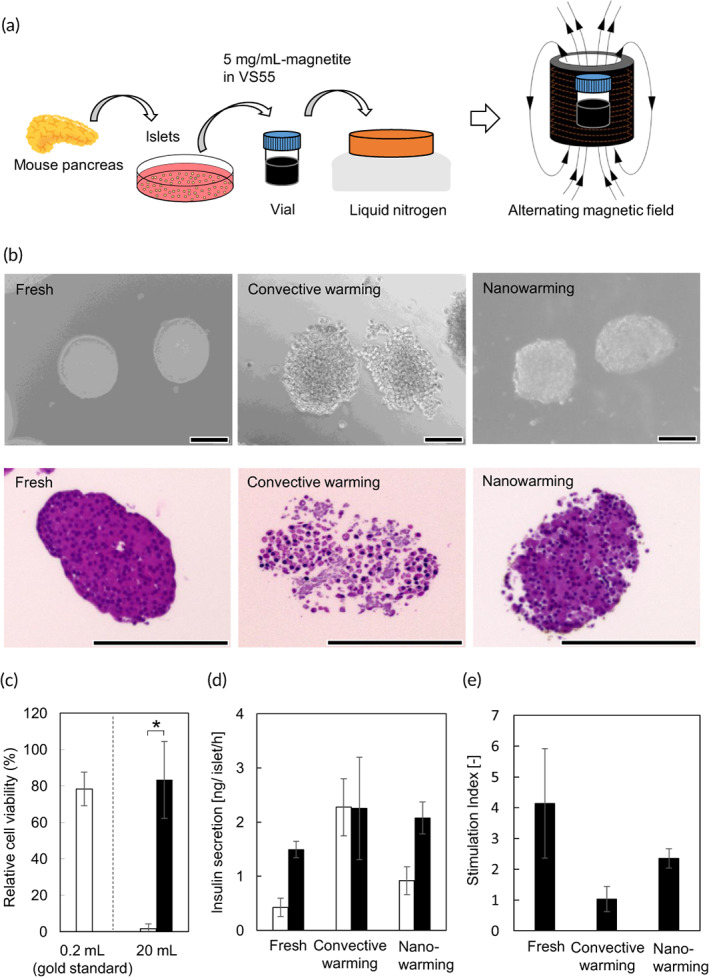
In vitro evaluation of nanowarmed islets. (a) Schematic illustration of nanowarming islets. Islets isolated from the mouse pancreas were precultured for 2 days and then added to mCPA (5 mg magnetite ml^−1^ VS55) in glass vials that were directly immersed in liquid nitrogen. Vitrified samples were then rewarmed by alternating magnetic field exposure of 10 kW at 208 kHz. (b) Morphology of islets. Bright‐field micrographs (top) and cross‐section (H&E staining) (bottom) of the islet. Left, freshly isolated islets; center, vitrified islets after rewarming by convective warming; right, vitrified islets after rewarming by nanowarming. Scale bars, 100 μm. (c) Effects of nanowarming on cell viability of islets. After rewarming vitrified islets (5 islets ml^−1^), cell viability was assayed using a cell viability imaging kit based on Hoechst 33342 for live cells and SYTOX green nucleic acid stain for dead cells. White columns, convective warming (water bath at 37°C); black column, nanowarming. Data are expressed as the mean ± SD of three independent experiments. Welch's *t*‐test was performed to compare the difference between the two groups. *p < 0.05. (d) Glucose challenge of islets. Mouse islets (20 islets per well) were subsequently incubated for 1 h in HKRB that contained 3 mM (white columns) and 20 mM (black columns) glucose. Insulin secretion (ng/islet/h) is expressed as the mean ± SD of triplicates. (e) Stimulation index. Data are expressed as the mean ± SD of three independent experiments.

Figure 4a shows the scheme of nanowarming islets. Pancreatic islets were isolated from mice, precultured for 2 days,^22^ vitrified in mCPA by submersion in liquid nitrogen, and nanowarmed by an alternating magnetic field for rewarming. To load and remove VS55, a slow stepwise dilution protocol^23^ at a low temperature (on ice) was used to minimize osmotic stress in the islets (Figure S2). Nanowarmed islets were applied to a cell strainer to remove magnetite nanoparticles prior to the slow stepwise dilution protocol. After the filtration and dilution, magnetite nanoparticles were not detected in nanowarmed islets. Also, we counted the number of nanowarmed islets after the filtration and dilution, and 81% ± 14% (*n* = 3) of islets were recovered from approximately 800 islets in 20 ml CPA. The morphology of islets after nanowarming was similar to that of freshly isolated islets, whereas cell–cell gaps were clearly observed in islets after convective warming (Figure 4b). Moreover, the Sytox green/Hoechst 33342 assay revealed that nanowarmed islets were highly viable, while dead cells were observed in vitrified islets after rewarming by convective warming (Figure S3). Also, nanowarmed islets showed both insulin and glucagon immunofluorescence staining similar to freshly isolated islets (Figure S4). Nanowarming of islets in 20 ml mCPA markedly improved cell viability (83.3% ± 21.0%) compared with convective warming (1.6% ± 2.7%), which was comparable to the 0.2‐ml sample (gold standard) (78.3% ± 9.3%) (Figure 4c). As a control, we also conducted a cryopreservation of islets with slow cooling (1 °C min^−1^) in 20 ml RPMI‐1640 medium supplemented with 10% DMSO. After rewarming by convective warming, cell viability of slow cooling was 8.8% ± 4.7%, which was significantly lower than that of fast cooling + nanowarming (Figure S5). As a mitochondrial marker, we examined ATP levels in islets after rewarming (Figure S6) and found that nanowarming maintained ATP content in islets better than convective warming (0.94 ± 0.14 vs. 0.06 ± 0.01, Figure S6). The glucose‐stimulated insulin secretion (GSIS) assay revealed that nanowarmed islets showed similar glucose‐stimulated insulin release to freshly isolated islets (Figure 4d). The stimulation index (SI) was calculated by dividing insulin levels in 20 mM glucose by those in 3 mM glucose (Figure 4e). The SI of freshly isolated islets was 4.1 ± 1.8, whereas that of nanowarmed islets was 2.4 ± 0.3. Conversely, vitrified islets after rewarming by convective warming showed high insulin levels at both low‐ and high‐glucose conditions (Figure 4d) with a low SI (1.0 ± 0.4) (Figure 4e), suggesting the unregulated insulin release caused by cellular damage due to cryopreservation.

**FIGURE 5:**
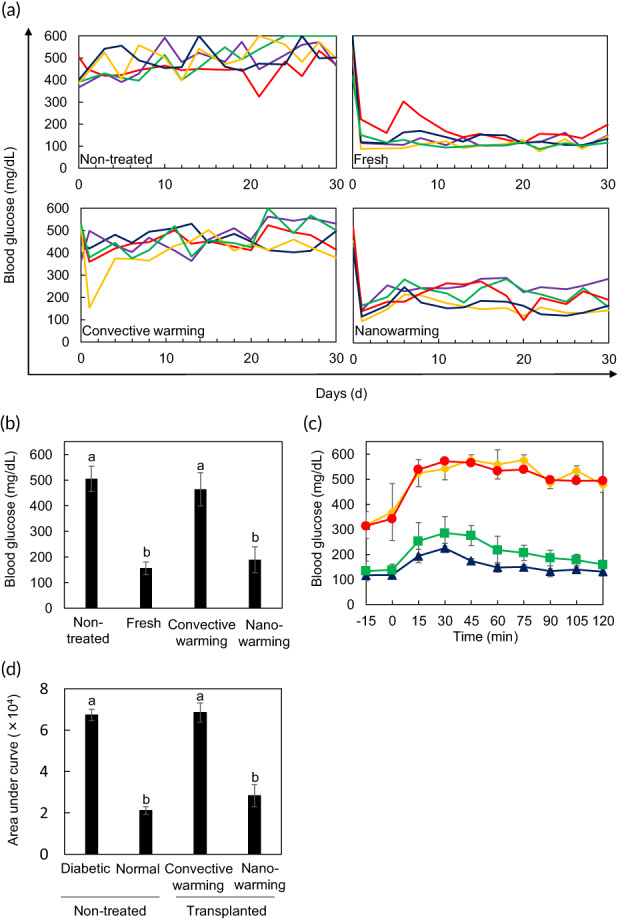
In vivo evaluation of nanowarmed islets. (a) In vivo transplant bioassay. Eight‐hundred islets were vitrified and rewarmed in 20 ml. Islets were transplanted into streptozotocin‐induced diabetic mice under the kidney capsule. After transplantation, the level of serum glucose was measured every 2 or 3 days. Each line represents the blood glucose concentration of each mouse (*n* = 5). Nontreated, untreated diabetic mice; fresh, diabetic mice transplanted with 400 freshly isolated islets; convective warming, diabetic mice transplanted with 400 convectively warmed islets; nanowarming, diabetic mice transplanted with 400 nanowarmed islets. (b) Blood glucose levels on Day 30 in (a). Data are expressed as the mean ± SD (*n* = 5). Comparisons between multiple groups were performed by Welch's *t*‐test with Bonferroni's correction. Different letters represent significantly different groups (*p* < 0.05). (c) Intraperitoneal glucose tolerance test (IPGTT) at 4 weeks after transplantation. Red circles, untreated diabetic mice; blue triangles, untreated normal mice; yellow diamonds, mice transplanted with convectively warmed islets; green squares, mice transplanted with nanowarmed warmed islets. Data are expressed as the mean ± SD. (d) Area under the curve (AUC) of IPGTT. Data are expressed as the mean ± SD (*n* = 5). Comparisons between multiple groups were performed by Welch's *t*‐test with Bonferroni's correction. Different letters represent significantly different groups (*p* < 0.05).

An in vivo transplant bioassay recognized as the gold standard of functional evaluation was conducted to assess the feasibility of clinical transplantation of nanowarmed islets (Figure 5a). Diabetic mice with nonfasting blood glucose of ≥300 mg dl^−1^ were established by STZ administration and untreated diabetic mice showed hyperglycemia for 30 days. Transplants of 400 freshly isolated islets lowered the blood glucose level to the normal level (≤200 mg dl^−1^). The nanowarmed islets (400 picked islets after nanowarming) successfully lowered blood glucose levels, whereas islets rewarmed by convective warming (400 picked islets after convective warming) did not achieve euglycemia. Figure 5b shows the blood glucose levels at 30 days after islet transplantation. The blood glucose level of mice treated with nanowarmed islets was comparable to that of mice treated with freshly isolated islets and was significantly lower than that of mice treated with islets rewarmed by convective warming. At 4 weeks after transplantation, an intraperitoneal glucose tolerance test (IPGTT) was performed (Figure 5c). Intraperitoneal injection of a glucose solution elevated the blood glucose levels in untreated diabetic mice, whereas in the normal mice, the elevated blood glucose values (highest peak at 30 min) were decreased to the normal level within 120 min. For vitrified islets in 20 ml, mice transplanted with nanowarmed islets successfully regulated the elevated blood glucose level (highest peak at 30 min), whereas mice treated with islets rewarmed by convective warming did not have lowered glycemic values. Figure 5d shows the area under the curve (AUC). The AUC of normal mice (21,088 ± 1932) and nanowarmed islets (28,290 ± 5352) was significantly lower than that of mice transplanted with islets rewarmed with convective warming (68,438 ± 4568) and untreated diabetic mice (67,290 ± 2753). These results indicate that nanowarmed islets are functional and effective for transplantation.

## 3 DISCUSSION

Cryopreservation should be an effective method for storing and pooling of donor islets for transplantation. As well as other tissues and organs, islets are complex structures and difficult to cryopreserve due to differential ice crystal formation. As a possible approach, researchers have proposed the methodology that islets are dissociated into single cells, cryopreserved as individual cells, and then reaggregated into their natural spherical form.^18^, ^24^ For mouse islet cryopreservation, the cell viability in a volume of 0.2 ml after rewarming by convective warming (gold standard) was approximately 80% of the fresh islets. The decrease in cell viability was likely due to toxicity from VS55. Recently, Zhan et al. achieved high recovery, viability, and function of islets by optimization of CPA composition (22% ethylene glycol + 22% DMSO) and CPA loading/unloading conditions.^25^ In the study, they also achieved high viability of islets by copper dish cooling and laser nanowarming of the vitrified droplet. Laser nanowarming, however, has volume limitation, and they used a thin cryomesh to achieve successful scale‐up in islet cryopreservation. A possible approach is thus scaling in the *x* and *y* dimensions of unidimensional devices including the cryomesh. Also, relatively thin containers can be scaling in the *x* and *y* dimensions. Lakey et al. successfully achieved bulk cryopreservation of canine islets by using a 500‐ml blood freezer bag.^26^ In this study with the aim of scale‐up of islet cryopreservation for biobanking and transplantation, we have shown the feasibility of nanowarming as a three‐dimensionally (in the *x*, *y*, and *z* dimensions) scalable technology for islet cryopreservation by physical (characterization of nanowarming rates) and biological (evaluation of nanowarmed islets) demonstrations. Specifically, we demonstrated that nanowarming achieved uniform and rapid rewarming of vitrified islets in volumes up to 20 ml. Moreover, we compared the functions of nanowarmed islets with the conventional method and demonstrated that nanowarmed islets showed improved viability of cells, GSIS, and the capability to lower blood glucose levels after transplantation. As compared with freshly isolated islets, on the other hand, nanowarmed islets showed a somewhat damaged morphology (Figure 4b), a lower SI in GSIS (Figure 4e), and slightly low capability to control blood glucose levels (Figure 5a,c), which was likely to be caused by damage due to cryopreservation using VS55. In the present study, 400 of nanowarmed islets were needed to lower the blood glucose levels in diabetic mice (Figure 5a,b), whereas researchers reported that freshly isolated 250 islets could lower the blood glucose levels.^24^ The relative islet potency following cryopreservation was thus estimated to be approximately 0.6 (250/400 for freshly isolated islets/nanowarmed islets), which was consistent with that based on the SI (2.4/4.1 for freshly isolated islets/nanowarmed islets) in GSIS (Figure 4e). That is, 1.6 of nanowarmed islets would be needed to replace one freshly isolated islet. Recently, Marquez‐Curtis et al. reported a single‐cell transcriptome study to identify differentially expressed genes between fresh and cryopreserved human islet cells.^27^ Further study including transcriptome analysis is needed to elucidate the mechanism of reduced functionality in islets nanowarmed with VS55.

In addition to intracellular ice formation that leads to cell death, intercellular ice formation impairs the integrity of multicellular tissues, and ice‐free cryopreservation by vitrification is crucial for long‐term biobanking of tissues and organs for transplantation. A mixture of cryoprotectants instead of one cryoprotectant in a slow‐freezing method solidifies at extremely low temperatures (glass transition temperature of −120 to −130°C) without ice formation. As a model CPA in the present study, we used VS55 composed of DMSO, formamide, and propylene glycol in Euro‐Collins solution with 55% (w/v) cryoprotectant. Magnetite nanoparticles were mixed with VS55 to prepare mCPA. DMSO has high diffusivity and enhances penetration across cell membranes.^28^ However, DMSO could exert a membrane thinning effect by downregulating cholesterol homeostasis genes, and systemic side effects have been also reported such as nausea, vomiting, nausea, vomiting, diarrhea, hemolysis, rashes, renal failure, hypertension, bradycardia, pulmonary edema.^29^ Dolezalova et al. reported that DMSO causes necrotic cell death at the periphery of islets.^30^ Nakayama et al. developed a CPA called “StemCell Keep” that contains nonpermeable carboxylated ε‐poly‐L‐lysine by replacement of permeable DMSO and showed improvement of rat islet viability after cryopreservation.^31^ Different from the traditional islet cryopreservation using a high concentration of permeable CPA, Cheng et al. developed a hydrogel‐based islet cell cryopreservation system by combination with nano‐delivery of 0.4 M trehalose.^32^ These approaches may obviate the need for any permeable CPA and thus avoid CPA toxicity. In the present study, to prepare mCPA, magnetite nanoparticles were well dispersed in VS55 and aggregation of magnetite was not observed at least for 1 h after preparation. In general, magnetite nanoparticles likely form aggregates in solutions other than water (i.e., buffer, culture medium, and organic solutions) and the composition of mCPA should be explored before use in nanowarming. Developing clinically available mCPA specialized for nanowarming is very important. To improve the stability of magnetite nanoparticles in CPA for nanowarming, Gao et al. developed microporous silica‐coated magnetite nanoparticles with a surface modified by PEG.^33^ Because PEG has also been used for a drug delivery system of magnetite nanoparticles in cancer hyperthermia,^34^ the PEGylation technology can be applied to nanowarming. For clinical applications, carboxydextran magnetite (Resovist),^35^ which is clinically available for magnetic resonance imaging, is also an attractive tool for nanowarming. Influences of chemically modified magnetite nanoparticles in CPA, such as interactions among nanoparticles, CPA, and target tissues/organs, remain to be investigated to develop new mCPAs specific for nanowarming.

Vitrification allows cryopreservation of tissues and organs indefinitely in theory, which may overcome the geographical problem of transplantation. In the present study, similar to the previous study on cryopreservation of human induced pluripotent stem cells,^9^ we conducted a trial in which mouse islets (20 ml mCPA) were vitrified in Kyoto University (Kyoto, Japan) and transported to Nagoya University (Aichi, Japan) (>100 km) at an ultralow temperature (≤−150°C) using a dry shipping container (cat# DR‐2DS, Cryo One, Osaka, Japan). As a result, the cell viability of islets vitrified in Kyoto University and nanowarmed in Nagoya University was comparable to that of islets both vitrified and nanowarmed in Nagoya University. Vitrification is thus robust and stable, and the major factors required for successful vitrification of tissues and organs are the cooling rate and storage temperature. For example, VS55 needs the cooling rate to exceed a CCR of 2.5°C min^−1^ and the storage temperature to be below the glass transition temperature of −123°C.

While vitrification has been practically used for cryopreservation of embryonic stem cells^36^ and embryos^5^ in CPA with small volumes, rewarming of large‐volume samples has remained challenging, which is related to the dual needs for the warming rate and uniformity. For example, warming rates faster than a CWR of 50°C min^−1^ and thermal gradients of ≤38°C are required for successful rewarming of VS55 to avoid damage caused by ice formation and cracking, respectively.^11^, ^21^ To achieve successful rewarming, nanowarming is advantageous over the conventional rewarming technique by convective warming. The nanowarming rates depended on the magnetic nanoparticle concentration (Figure 2a), power output (Figure 2b), and frequency (Figure 2c). The quantity of heat generation by magnetic nanoparticles is proportional to the frequency and the square of the magnetic field strength.^13^ The mechanisms of magnetic heating of superparamagnetic nanoparticles are Néel relaxation and Brownian relaxation. Néel relaxation is derived from reorientation of the magnetic moment in the same direction as the applied magnetic field with each field oscillation.^37^ Brownian relaxation is caused by the friction that arises from the rotation of the particle in the carrier liquid.^38^ Because CPA is highly viscous, the dominant mechanism of heat dissipation may be Néel relaxation. Further study is needed to elucidate the mechanism of heat dissipation of magnetic nanoparticles in CPA.

In order to minimize toxicity from CPA exposure and osmotic shock, CPA needs to be carefully loaded and removed from biologic systems. In the present study, a slow stepwise dilution protocol^23^ was used to mitigate osmotic damage in the islets (Figure S2). By combining with filtration of nanowarmed islets, magnetite nanoparticles were not detected in nanowarmed islets after the slow stepwise dilution. Researchers have reported image‐guided characterization of mCPA distribution in organs/tissues by microcomputed tomography imaging,^8^, ^11^ magnetic resonance imaging,^8^ and magnetic particle imaging.^10^ These imaging technologies will be a potent tool to better guide the loading and unloading process.

In the present study, we succeeded in nanowarming 20 ml mCPA that contained 800 islets. Clinically, in the Edmonton protocol, at least 10,000 islet equivalent (IEQ) per 1 kg of body weight is required for a transplant. A human pancreas has ~1 × 10^6^ islets, but successful islet isolation is reported to be 1 × 10^5^ to 3.5 × 10^5^ IEQ per donor,^39^ which indicates that two or three donors are required for a transplant. For example, if a 60 kg patient needs 6 × 10^5^ IEQ, a system capable of cryopreserving 2 × 10^5^ IEQ from three donors each is required. Taylor and Baicu proposed a vitrification protocol in which the islet concentration in CPA is 500 IEQ ml^−1^.^17^ Therefore, the CPA volume for 2 × 10^5^ IEQ is estimated to be several hundred milliliters and scale‐up of the heating coil is necessary for clinical application. In the present study, a coil with a diameter of 70 mm and a height of 80 mm was used for nanowarming. We previously prototyped a coil with a diameter of 300 mm for clinical cancer hyperthermia. However, in cryopreservation, samples with a large diameter will not vitrify because of the slow cooling rate even with the use of liquid nitrogen. For VS55, a rough estimation suggests that samples with a diameter of <50 mm can achieve the CCR (2.5°C min^−1^). However, during rewarming, we demonstrated that nanowarming resulted in uniform warming and the warming rates were independent of the sample volume (Figure 3b). For scale‐up, a long (in height) cylindrical coil has been considered to be a possible design for a clinical applicator of an alternating magnetic field.

## 4 MATERIALS AND METHODS

### 4.1 Study design

The aim of this study was to evaluate the efficacy of nanowarming to rewarm islets after vitrification compared with conventional rewarming by convective warming. We first conducted experiments using the conventional method by rapid cooling in liquid nitrogen and rewarming by convective warming in a 37°C water bath to demonstrate the size limitation caused by CWR in rewarming using 1–30 ml CPAs. The glass vials used in this study are shown in Table S1. Additionally, 0.2 ml CPA in 2‐ml polypropylene cryovials (Corning, New York, NY) was used as a control (gold standard). We then conducted the physical characterization of nanowarming using mCPA without islets. By changing parameters (concentration of magnetite nanoparticles in CPA, power output, and frequency), we found the optimal nanowarming conditions that were sufficiently uniform and rapid to exceed CWR. Using the optimized conditions, an in vivo transplant bioassay as the gold standard of functional evaluation was conducted to assess the feasibility of clinical transplantation of nanowarmed islets. Freshly isolated and convectively rewarmed islets were used as positive and negative controls, respectively. The number of vials, islets, and mice used in each experiment and replicate numbers of each experiment are included in the figure captions. All animal experiments were approved by the Ethics Committee for Animal Experiments of the School of Engineering, Nagoya University (G210480).

### 4.2 VS55 and magnetite nanoparticles

VS55 is a CPA that contains 3.1 M DMSO (Fujifilm Wako Chemical, Osaka, Japan), 3.1 M formamide (Fujifilm Wako Chemical), and 2.2 M propylene glycol (Fujifilm Wako Chemical) in Euro‐Collins solution with 55% (w/v) cryoprotectant.^21^ The magnetite nanoparticles (Fe_3_O_4_; average particle size: 10 nm) were obtained from Dai‐ichi High Frequency (Tokyo, Japan). The magnetic characteristics at 796 kA m^−1^ (room temperature) were as follows: 2.0 kA m^−1^ coercivity, 63.9 Am^2^ kg^−1^ saturation flux density, and 2.6 Am^2^ kg^−1^ remanent flux density. To prepare mCPA, the magnetite nanoparticles were mixed with VS55 at the indicated concentrations. The mCPA was sonicated for 60 min and vortexed for 5 min before use.

### 4.3 Tissue culture

Pancreatic islets were isolated from adult male (6–8 weeks old) Slc:ddY mice (Japan SLC, Shizuoka, Japan) by a collagenase digestion technique.^25^ Mice were sacrificed by neck dislocation and midline laparotomy was performed. A clamp (Muromachi Kikai, Tokyo, Japan) was placed on the duodenal papilla to prevent the perfusate from flowing out into the duodenum. A 3 ml of 0.5 mg ml^−1^ collagenase solution (collagenase P from *Clostridium histolyticum* [Sigma‐Aldrich, Saint Louis, MO] in Hank's balanced salt solution) was slowly injected into the pancreas via the bile duct with a 30 G needle. The swollen pancreas was then carefully removed from the mice, collected in a 50 ml centrifuge tube containing 2–3 ml of collagenase solution, and kept on ice until the pancreas of all mice were isolated. Tubes were incubated in at 37°C in a water bath and shaken every 5 min until the pancreas was digested (12–18 min). To stop digestion, 10 ml of cold KRB buffer supplemented with 1 mM CaCl_2_ were added to into each tube and undigested impurities such as fat were removed. After further adding KRB buffer + CaCl_2_ to mass up the liquid volume to 45 ml, the supernatant was removed by centrifugation at 370 *g* for 30 s at 4°C. After one more washing with KRB buffer + CaCl_2_, the digest was resuspended in 3 ml Histopaque‐1119 (Sigma‐Aldrich) at room temperature. The suspension was carefully transferred into a siliconized glass tube, and 2 ml Histpaque‐1077 (Sigma‐Aldrich) and 2 ml Histpaque‐1050 (Sigma‐Aldrich) were layered on the top. The tube was then centrifuged at 440 *g* for 10 min without brake. The islet‐enriched layer between Histpaque‐1077 and Histpaque‐1050 was collected into a 100 mm Petri dish containing KRB buffer + CaCl_2_. Islets were picked up by a pipette and immediately transferred for culture. Islets were cultured in suspension in tissue culture dishes that contained RPMI 1640 medium supplemented with 10% fetal calf serum, 0.1 mg ml^−1^ streptomycin sulfate, and 100 U ml^−1^ potassium penicillin G at 37°C in a humidified atmosphere with 5% CO_2_.

### 4.4 Nanowarming

Nanowarming of vitrified samples was performed as reported previously.^9^ Briefly, an alternating magnetic field (31.9 kA m^−1^) was created using a vertical coil (inner diameter: 70 mm; length: 80 mm) with a transistor inverter (HI‐HEATER6020; Dai‐ichi High Frequency) operating at 108, 163, or 208 kHz. Temperatures during exposure of the alternating magnetic field were monitored by optical fiber probes (FL‐2000; Anritsu Meter, Tokyo, Japan). To remove magnetite nanoparticles, nanowarmed islets were applied to a cell strainer (pore size, 40 μm; Corning).

### 4.5 Cell viability assay

Immediately after rewarming, cell viability was assayed using a ReadyProbes Cell Viability Imaging Kit (Blue/Green) based on Hoechst 33342 for live cells and SYTOX green nucleic acid stain for dead cells (Thermo Fischer Scientific, Waltham, MA).^9^ For mouse islets, tissues were dissociated into single cells using Accumax (Innovative Cell Technologies, San Diego, CA, USA). Cell viability was determined as the percentage of live cells among total cells counted in five fluorescence images of each sample under a fluorescence microscope (BZ‐X810; Keyence, Tokyo, Japan). In some cases of mouse islets, the relative cell viability was determined by the following equation:

$$\text{Relative cell viability}\;\left(\%\right)=\frac{\text{cell viability of the sample}}{\text{cell viability of freshly isolated islets}}\times 100.$$

### 4.6 Insulin secretion assay

The GSIS assay was carried out to assess the beta cell function. Briefly, 20 mouse islets per group were incubated in HEPES‐balanced Krebs‐Ringer bicarbonate (HKRB) buffer that contained 3 mM glucose for 1 h at 37°C in a humidified atmosphere with 5% CO_2_. The tissues were then stimulated with HKRB buffer that contained 20 mM glucose by replacing the buffer and incubated again for 1 h. At the end of each incubation, supernatants were collected and stored at −80°C until the assay. Basal (3 mM glucose) and stimulated (20 mM glucose) insulin levels were determined with an Ultra‐Sensitive Mouse Insulin ELISA kit (Morinaga Institute of Biological Science, Kanagawa, Japan) in accordance with the manufacturer's instructions and the SI was determined by dividing the stimulated insulin level by the basal insulin level.

### 4.7 Histology

Mouse islets were washed three times with phosphate‐buffered saline (PBS), fixed in 4% paraformaldehyde in PBS, and embedded in paraffin. Thin sections (4 μm) were prepared, stained with hematoxylin and eosin, and then observed under a BZ‐X810 microscope (Keyence).

### 4.8 In vivo transplant bioassay

Diabetes was induced by single‐dose (180 mg kg^−1^) intraperitoneal administration of STZ (Sigma‐Aldrich) into KSN/Slc athymic mice (Japan SLC) 7 days preoperatively. The blood glucose level of mice was determined using an Experimental Animal Glucometer SUGL‐001 (ForaCare, Tokyo, Japan) by tail vein bleeds. Blood glucose levels were measured after 2–7 days, and mice with nonfasting blood glucose levels of over 300 mg dl^−1^ in two consecutive measurements were defined as having diabetes. Under general anesthesia with isoflurane inhalation solution (Mylan, Pittsburgh, PA), mouse islets immediately after rewarming were transplanted into diabetic mice under the recipient mouse's left kidney capsule. For all treatment groups, islets were randomly selected for transplantation, including islets of all sizes, both morphologically disrupted and intact islets.^25^ The serum glucose of the mice was measured every 2 or 3 days throughout the 4‐week observation period.

An IPGTT was performed after overnight fasting at 4 weeks after transplantation. The mice were fasted for 16 h and then a glucose solution (1 g in 10 ml kg^−1^ body weight) was injected intraperitoneally. Blood glucose levels were measured before (−15 min) and every 15 min for 120 min after injection. The AUC of IPGTT was also calculated.

### 4.9 Statistical analysis

Welch's *t*‐test was used to compare differences between two groups. Comparisons between multiple groups were performed by Welch's *t*‐test with Bonferroni's correction. Differences were considered statistically significant at *p* < 0.05.

## 5 CONCLUSIONS

In the present study, we have demonstrated that nanowarmed islets function to release insulin in response to glucose stimulation and successfully lower the blood glucose levels of STZ‐induced diabetic mice after transplantation. Before entering clinical application, a preclinical study using human islets from deceased donors should be conducted. Jutte et al. reported that human islets appeared to withstand vitrification very well compared with mouse islets,^20^ which suggests that the results obtained from mouse islets in the present study are promising. Taken together, our results suggest that ice‐free cryopreservation by nanowarming will lead to breakthroughs in biobanking of islets for transplantation.

## AUTHOR CONTRIBUTIONS

**Taisei Wakabayashi:** Data curation (equal); investigation (lead); methodology (lead); writing – original draft (equal); writing – review and editing (equal). **Masahiro Kaneko:** Project administration (equal); supervision (equal); validation (equal); writing – original draft (equal); writing – review and editing (equal). **Tomoki Nakai:** Investigation (equal); methodology (equal). **Masanobu Horie:** Conceptualization (equal); funding acquisition (supporting); investigation (equal); methodology (equal); writing – review and editing (equal). **Hiroyuki Fujimoto:** Investigation (equal); methodology (equal); writing – review and editing (supporting). **Masazumi Takahashi:** Investigation (supporting); methodology (equal); writing – review and editing (supporting). **Shota Tanoue:** Investigation (supporting); methodology (equal); writing – review and editing (supporting). **Akira Ito:** Conceptualization (lead); funding acquisition (lead); investigation (equal); methodology (supporting); project administration (lead); supervision (lead); validation (lead); writing – original draft (lead); writing – review and editing (lead).

## CONFLICT OF INTEREST

The authors declare that they have no known competing financial interests or personal relationships that could have appeared to influence the work reported in this paper.

### PEER REVIEW

The peer review history for this article is available at https://publons.com/publon/10.1002/btm2.10416.

## Supporting information


**Appendix S1:** Supporting Information

## Data Availability

The data that support the findings of this study are available from the corresponding author upon reasonable request.
