# Supplementary material for: Nanowarming of vitrified pancreatic islets as a cryopreservation technology for transplantation
Source: Bioeng Transl Med. 2022 Sep 27;8(4):e10416. doi: 10.1002/btm2.10416 (PMC10354753; doi:10.1002/btm2.10416)
Supplement: Supplementary file 1 — Appendix S1: Supporting Information [file BTM2-8-e10416-s001.pdf]

Supplementary Information

Nanowarming of vitrified pancreatic islets as a cryopreservation technology for transplantation

Taisei Wakabayashi[1] | Masahiro Kaneko[1] | Tomoki Nakai[1] | Masanobu Horie[2] | Hiroyuki Fujimoto[2] | Masazumi Takahashi[3] | Shota Tanoue[3] | Akira Ito[1]

[1]Department of Chemical Systems Engineering, School of Engineering, Nagoya University, Nagoya, Japan

[2]Radioisotope Research Center, Agency of Health, Safety and Environment, Kyoto University, Kyoto, Japan

[3]Technical Department, Dai-Ichi High Frequency Co., Ltd. Kawasaki, Japan

Correspondence

Akira Ito, Department of Chemical Systems Engineering, School of Engineering, Nagoya University, Furo-cho, Chikusa-ku, Nagoya 464-8603, Japan.

Email: ito.akira@material.nagoya-u.ac.jp

This Additional file includes:

Methods
Figures S1–S6
Table S1

Methods

Slow cooling of mouse islets. Islets (5 islets/mL) were added into a 20-mL vial containing RPMI-1640 medium supplemented with 10 % DMSO for 20 min at room temperature and for 30 min in ice bath. The vials were placed in a freezing container (Mr. Frosty; Thermo Fischer Scientific) and cooled in a freezer at −80 °C overnight. Convective warming for thawing was carried out by immersing the vials in a 37 °C water bath. Cell viability was assayed using a cell viability imaging kit based on Hoechst 33342 for live cells and SYTOX green nucleic acid stain for dead cells.

Immunostaining. Immunostaining. Islets were fixed in 10% formalin and embedded in paraffin blocks. Then, thin sections (5 μm thick) were deparaffinized using lemozol (FUJIFILM Wako) and decreasing 100%, 90%, and 70% ethanol concentrations and heated for 5 min at 90 °C in PBS for antigen-retrieval. Slides were incubated with blocking buffer (3% bovine serum albumin (Nacalai tesque) in PBS) for 30 min and then incubated with rabbit polyclonal anti-insulin antibody (H-86; Santa Cruz Biotechnology) (1:200) and mouse monoclonal anti-glucagon antibody (K79bB10; Santa Cruz Biotechnology) (1:200) overnight at 4°C. The sections were then washed with PBS and incubated with Alexa Fluor 488 goat anti-rabbit IgG (A-11034; Thermo Fisher Scientific) (1:100) and Alexa Fluor 546 goat anti-mouse IgG (A-11030; Thermo Fisher Scientific) (1:200) for 1 h at room temperature. After washing with PBS, the specimens were observed under a fluorescence microscope (BZ-X710; Keyence).

ATP measurement. Islets were incubated in a 100 mm tissue culture dish at 37 °C and 5% CO_2 for 3 h before the assay. Five islets per condition were handpicked and dissolved in ATP assay regent (TOYO B-Net, TA100). A Lumat LB 9507 (Berthold) was used to measure the luminescence.

TABLE S1 Glass vials used in this study.

Name of system	Inner diameter (mm)	Outer diameter (mm)	Height (mm)	Solution volume (mL)
1 mL	10.6	12	35	1
8 mL	19	21	45	8
20 mL	27.6	30	63	20
30 mL	32.4	35	78	30

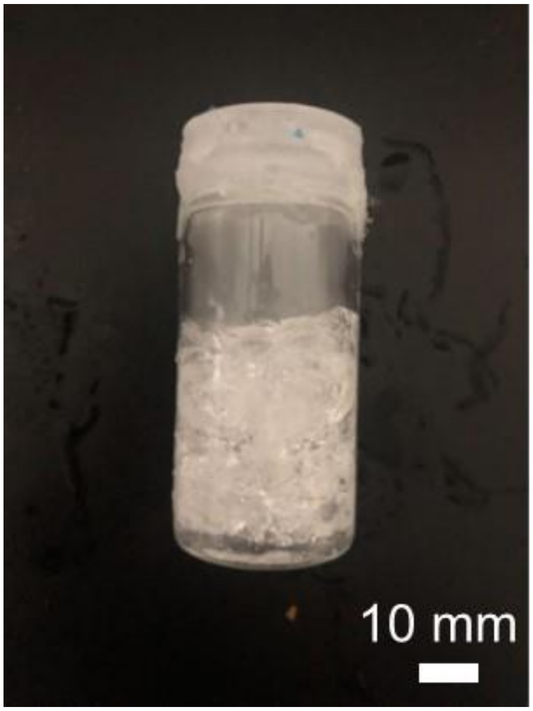

FIGURE S1 Crack formation during rewarming by conductive warming. A representative photo of a 20-mL sample is shown.

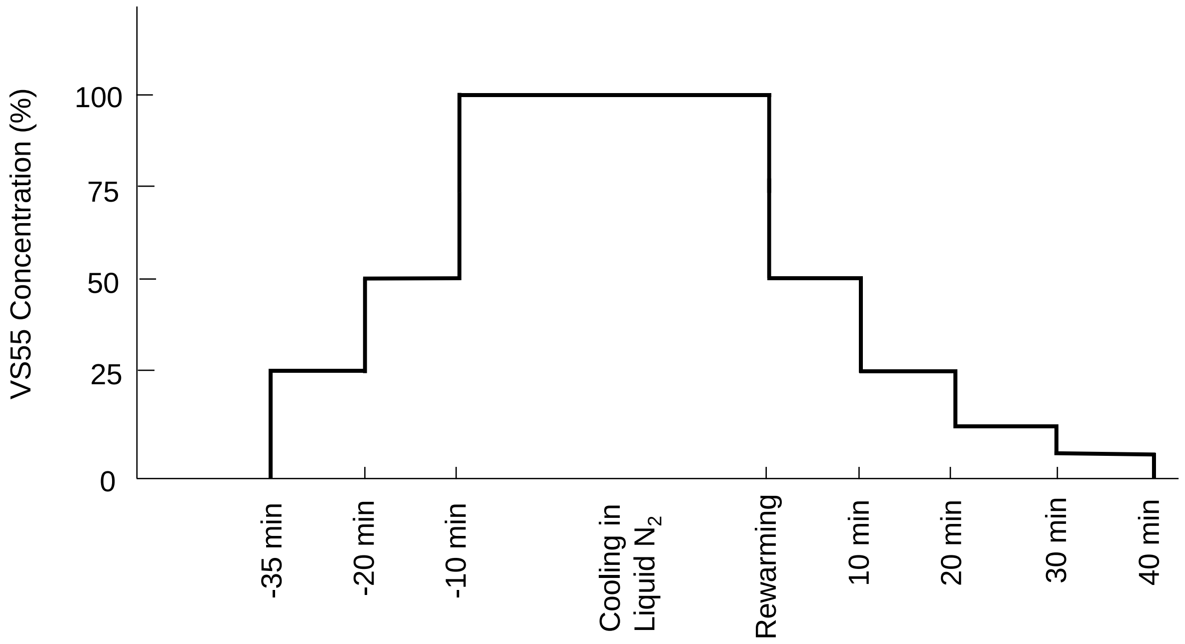

FIGURE S2 Schematic of the slow stepwise dilution protocol. Changes in the VS55 concentration over time are shown.

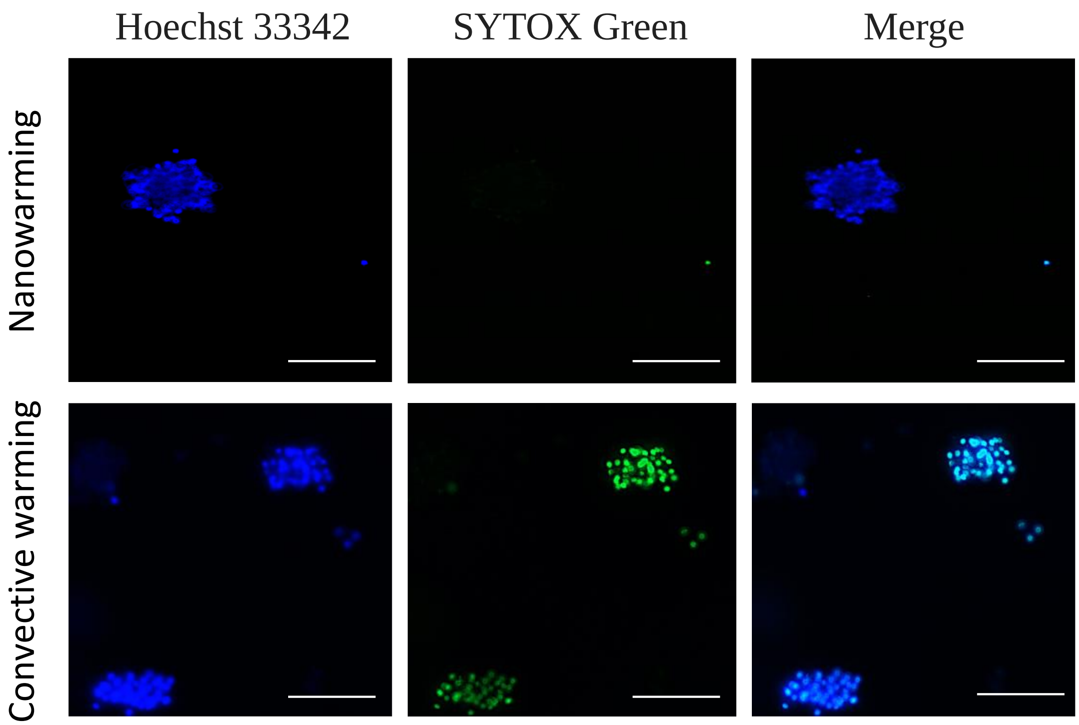

FIGURE S3 Effects of nanowarming on the viability of mouse islets. Hoechst 33342 was used as a cell-permeable DNA labeling blue dye. The green fluorescence dye SYTOX Green, which cannot cross intact cell membranes, was used to stain dead cells. Scale bars, 200 μm.

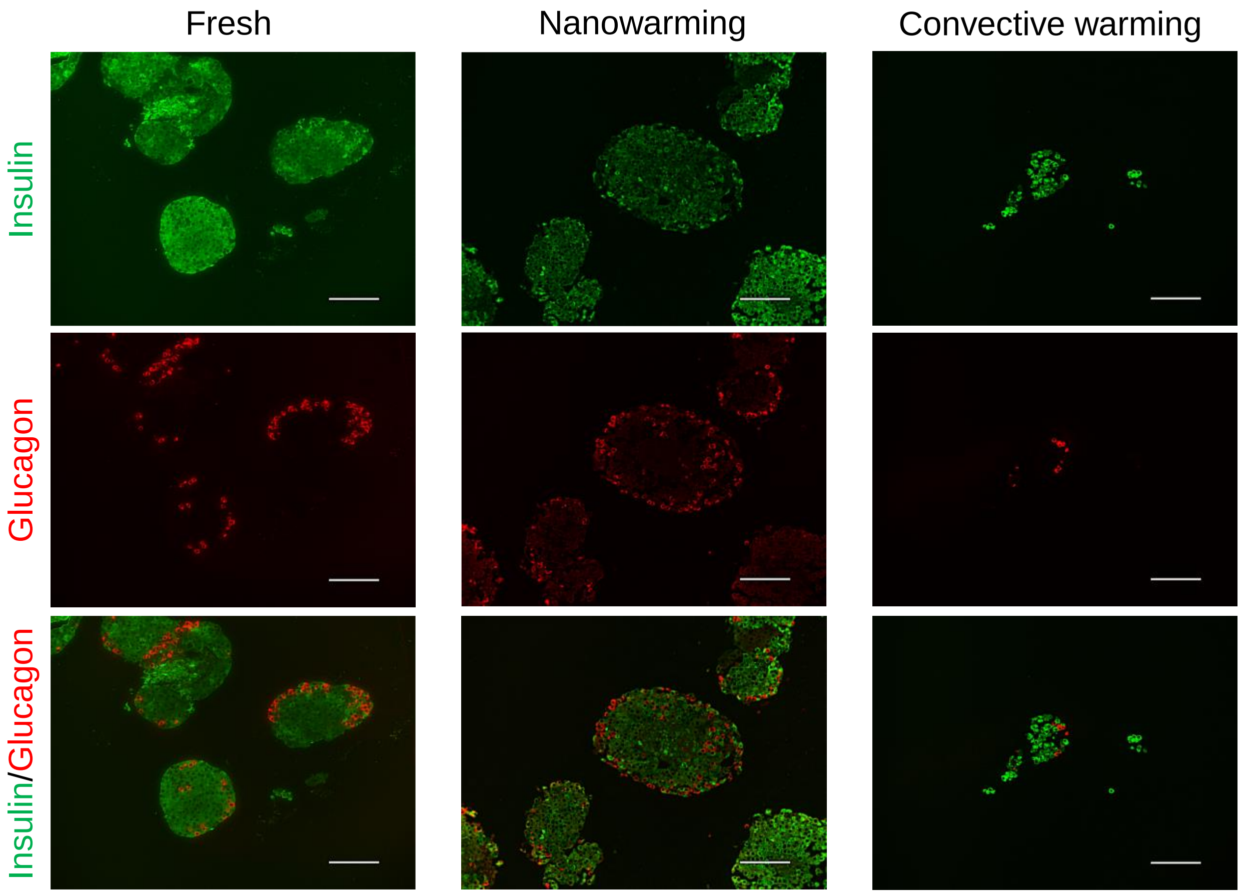

FIGURE S4 Insulin (green) and glucagon (red) staining in freshly isolated (Fresh), nanowarmed (Nanowarming) or convective-warmed (Convective warming) islets. Scale bar, 100 µm.

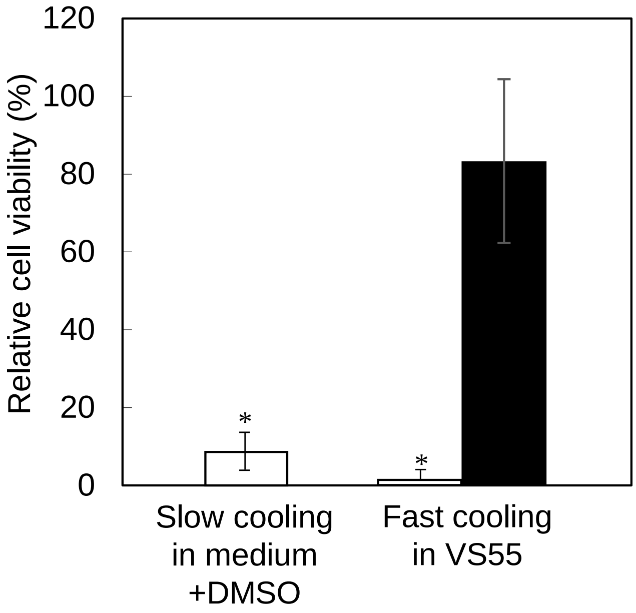

FIGURE S5 Comparison of islet cryopreservation with slow cooling and fast cooling. For slow cooling, islets (5 islets/mL) were added into a 20-mL vial containing RPMI-1640 medium supplemented with 10 % DMSO for 20 min at room temperature and for 30 min in ice bath, and the vial was cooled at 1°C/min to reach -80°C. For fast cooling, 20-mL vials were submersed in liquid nitrogen. After rewarming cryopreserved islets, cell viability was assayed using a cell viability imaging kit based on Hoechst 33342 for live cells and SYTOX green nucleic acid stain for dead cells. White columns, convective warming (water bath at 37°C); black column, nanowarming. Data are expressed as the mean ± SD of three independent experiments. Welch's t-test was performed to compare the difference between the two groups. *$p < 0.05$ vs nanowarming.

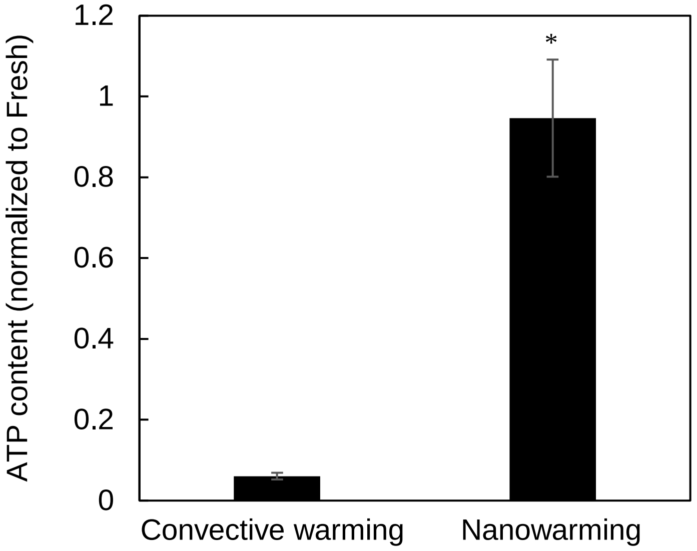

FIGURE S6 ATP content of cryopreserved islets 3 h post-thaw normalized to ATP content in freshly isolated islets. Data are expressed as the mean ± SD of three independent experiments. Welch's t-test was performed to compare the difference between the two groups. *p < 0.05.
